# *Announcement: *Community Preventive Services Task Force Recommendation for Family-Based Interventions to Increase Physical Activity

**DOI:** 10.15585/mmwr.mm6618a9

**Published:** 2017-05-12

The Community Preventive Services Task Force recently posted new information on its website: “Physical Activity: Family-Based Interventions.” This information is available at https://www.thecommunityguide.org/findings/physical-activity-family-based-interventions.

Established in 1996 by the U.S. Department of Health and Human Services, the task force is an independent, nonfederal, panel of public health and prevention experts whose members are appointed by the director of CDC. The task force provides information for a wide range of persons who make decisions about programs, services, and other interventions to improve population health. Although CDC provides administrative, scientific, and technical support for the task force, the recommendations developed are those of the task force and do not undergo review or approval by CDC.

